# Supplementary material for: Are carbon emissions trading and green financial instruments synergistic? -Comprehensive quantitative research based on content analysis
Source: PLoS One. 2024 Mar 7;19(3):e0298601. doi: 10.1371/journal.pone.0298601 (PMC10919601; doi:10.1371/journal.pone.0298601)
Supplement: S1 Data — (ZIP) [file pone.0298601.s001.zip › Data/地级市Z_F工作报告.zip!/╣π╬≈/╬α╓▌╩╨/└┤╫╘╣1⁄2╓┌║┼í╛┬φ┐╦╩2╛▌═°í┐_2019.docx]

各位代表：

现在，我代表市人民政府向大会报告政府工作，请予审议，并请市政协委员和列席会议的同志提出意见。

一、2018年工作回顾

2018年，我市面临的困难和挑战超出预期。在自治区党委、政府和市委的坚强领导下，我们以习近平新时代中国特色社会主义思想为指导，认真贯彻落实党的十九大和十九届二中、三中全会精神，坚持稳中求进工作总基调，坚持推动高质量发展，以“三年一工程”为抓手，毫不动摇实施“东融”战略，扎实做好稳增长、促改革、调结构、惠民生、防风险各项工作，推动全市经济社会平稳健康发展。全市地区生产总值增长1.8%，规模以上工业总产值下降4.3%，固定资产投资增长9.8%，财政收入增长0.6%，社会消费品零售总额增长9.5%，金融机构存款余额、贷款余额分别增长7.5%和10.4%，城镇居民、农村居民人均可支配收入分别增长6.3%和10.4%。虽然个别指标低于预期，但全市经济社会发展稳中提质、稳中有进，呈现良好的发展态势。

一批经济先行指标保持良好发展势头，支撑发展能力正在增强。工业用电量增长20%，工业投资增长25.7%，工业税收增长19.8%，财政收入质量提高；万元工业增加值能耗下降15.6%；各类市场主体增长25.47%，电子商务交易总额增长53%，限上批发业销售额增长27.7%；公路客货运周转量增长9%，水路货运周转量增长15.9%，长洲船闸过货量达1.32亿吨，比上年增长33.4%。

一批产业开工竣工、复产达产，工业运行质量和效益正在提升。国光梧州产业基地、生物医药产业园、岭南食品工业小镇（产业园）等开工建设。国龙15万吨PET再生塑料、顶峰30万吨不锈钢冷连轧、欧神诺陶瓷等项目竣工投产。金升30万吨高纯阴极铜成功重组，32家停产企业成功复产，全市近80%的重点企业实现稳定增长。

一批重点招商引资项目成功落地，新旧动能接续转换正在加快。梧州医学高等专科学校、昭衍（梧州）新药研究中心、梧州恒大养生谷、启迪鸿星（梧州苍海）冰雪文体小镇、国信优易大数据、蒙娜丽莎陶瓷等成功落户，“美的·中央广场”城市综合体、正威（梧州）国际宝玉石文化创意产业园、中盟中小企业园等加快推进。

一批重大交通项目取得突破进展，国内区域性综合交通运输枢纽地位不断巩固。梧州至信都高速公路、贵港至梧州3000吨级航道工程等开工建设，贺州至巴马、荔浦至玉林高速公路梧州段、西江四桥等加快推进，龙圩综合客运枢纽站主体工程竣工，市环城高速公路全线通车。梧州西江机场建成，并开通至北京、上海、长沙等城市航线，梧州由“高铁时代”迈向新的“航空时代”。

一批改革发展平台取得良好成效，改革发展活力不断释放。梧州港赤水作业区扩大开放通过国家验收，循环经济产业园区获批国家“绿色园区”，蒙山县被授予第二批国家生态文明建设示范县称号，在全国首创基本医保门诊特殊慢性病直接结算，我市社会保险费征管“四个率先”经验在全区推广，广西首张新版农村土地承包经营权证和首张个体工商户全程电子化营业执照在我市颁出。

一批群众多年关切的城建和社会事业项目顺利推进，城市承载力不断提高。第一污水处理厂提标改造工程圆满完工，静脉产业园餐厨垃圾处理厂正式投入运营，第三污水处理厂加快建设，市区饮用水取水点上移工程全面启动，塘源水厂取水点调整如期完成，市人民医院完成搬迁，棚户区改造工程取得重要进展，新兴、京梧、两广等农贸市场综合环境明显改观。撤销城北、城南、城西、龙圩等城市路桥收费站，打通银湖北路中段、新湖一路南段等“断头路”，一批困扰市民出行的“堵心路”变成人民群众的“顺心路”。

一年来，我们重点抓了以下工作：

（一）抓工业，稳增长。围绕做优一批产业、救活一批企业、引进一批项目，精准施策。安排1亿元财政资金，支持重点企业做大做强，推动优势产业升级改造，推进园区提档升级。帮助企业解决一批历史遗留问题和发展难题，降低企业成本18.04亿元，其中降低企业用电成本2.47亿元。“惠企贷”累计发放超过5亿元。新增规上企业47家，累计签约工业项目77个，引进亿元工业项目64个。大力实施质量强梧战略，授予3家企业市长质量奖。新组建4家自治区级企业工程技术研究中心，完成科技成果转化43项，同比增长115%，高新技术企业保有量达69家。

（二）抓三产，扩内需。深挖传统服务业潜力，邮政业务量增长24%，商品房销售面积增长40.1%。“互联网＋”新业态加快发展。苍海新区“康养+医养”项目、大数据项目加快推进，游客集散中心投入使用。着力推动旅游业提质发展，成功举办“2018北京国际茶业展·北京马连道国际茶文化展·梧州六堡茶文化节”、中国（梧州）六堡茶暨广西岭南风情文化旅游周活动，推动骑楼城、石表山风景区争创5A景区，（下转第八版）

（上接第一版）全力推进全域旅游示范区创建，蒙山县荣获第四批广西特色旅游名县。新增自治区旅游度假区和广西四星级汽车旅游营地各1家、广西生态旅游示范区2家、四星级以上乡村旅游区（农家乐）11家。全年接待游客突破3000万人次，旅游总消费增长42.2%。全市第三产业增加值完成469.85亿元，增长9.2%。

（三）抓“三农”，固根基。加快实施产业发展、基础设施和公共服务能力提升3个专项行动。深入推进“第一公里·路桥”惠民工程，建成农村道路344条、桥梁403座。新增创建各级现代特色农业示范区（园）78个。水果、蔬菜和茶叶种植面积分别达到84.82万亩、130万亩和10.01万亩。建成广西规模最大蛋鸡生产基地、单体最大生猪繁衍基地，蜂蜜产量稳居全区第一。新增农民合作社336家、家庭农场80家，发展名优品牌和“三品一标”产品79个。林业经济稳步发展，成功举办首届中国（梧州）林产工业发展高峰论坛。水稻生产综合机械化水平达到72%以上。成功举办首届农民丰收节。全市第一产业增加值增长5.9%，创近8年来新高。

（四）抓改革，促开放。推进26项重点改革任务，一批试点形成制度性成果。下好“放管服”改革先手棋，建立行政审批容缺受理、并联审批等工作机制，96.5%的行政审批事项实现“最多跑一次”，市本级98%的政务服务事项实现网上申办。完成农村土地确权登记颁证工作，统筹推进财政、电力、医药卫生等体制改革，将全市16家国有企业重组为4家国有集团公司。加快建设粤桂合作特别试验区，新增46家企业落户、8家企业上规入库、7家企业实现投产。全市完成招商引资区外到位资金722亿元，区外投资项目开工率达90%以上。成功举办第十五届梧州宝石节、西江经济带城市共同体及市长联席会议第三次会议暨西江经济发展论坛、大数据产业发展论坛、梧茂战略合作第三次市长联席会议。

（五）抓城建，优环境。统筹推进203个重点城建项目，实施道路贯通工程、“白改黑”工程，完成一批城市主次干道、公园广场改造提升，城市面貌发生喜人变化。苍海新区万亩湖面实现永久性蓄水，苍海小学、环湖道路等一批项目建成。棚户区改造开工6535套（户），保障性住房累计分配入住23838套。率先实行街长制、路长制，探索建立城市管理“十大负面清单”，大力整治城乡综合环境，城乡环境明显改善。加快西江明珠塔、新加坡印象等烂尾项目复工进程。加快推进数字梧州建设，28个部门55个系统实现信息共享，“桂民卡·梧州通”实现与全国270多个城市互联互通。创建1个国家级生态县、2个自治区级生态县、40个自治区级生态乡镇、55个自治区级生态村，岑溪市筋竹镇云龙村入选中国历史文化名村。积极开展乡村风貌提升三年行动，完成农村改厨、改厕各4.53万户，危房改造开工8351户。创建广西双拥模范城通过自治区考评。

（六）抓攻坚，破难题。落实财政专项扶贫资金9.91亿元，全力打好“五场硬仗”，改善贫困村基础设施，提高产业扶贫覆盖率，建设易地扶贫搬迁安置房933套、搬迁入住4040人，全市85个贫困村和苍梧县整县达到脱贫摘帽标准，预计实现56795人脱贫。打好碧水蓝天净土三大保卫战，狠抓中央环保督察“回头看”反馈问题整改，完成市级集中式饮用水水源地环境问题整治，与自治区生态环境厅建立厅市合作共建机制，首创工业园区“环保管家”第三方治理机制。空气质量优良率达到94.5%，比上年提升4.1个百分点；PM2.5年均浓度控制在自治区下达指标以内，比上年下降9.8%；集中式饮用水水源地水质达标率保持100%。做好政府存量债务置换，规范政府投融资行为，全市债务风险总体可控。

（七）抓民生，增福祉。11类民生实事全面完成，财政支出81.7%用于民生领域。城镇新增就业3.25万人，农村劳动力转移就业新增5.22万人次，城镇登记失业率为1.93%。农村义务教育学校建设项目、薄弱学校改造工程完成投资1.95亿元，基本消除超大班额、大通铺，全市义务教育巩固率达97.92%，排全区第二位；长洲区义务教育均衡发展通过国家验收，苍梧县、藤县义务教育均衡发展通过自治区督导评估；新建2所、改扩建17所普通高中。组建1个医疗集团、5个医联体、11个医共体，开通全国首个基于医疗物联网的家庭医生签约服务平台。残疾人两项补贴全面落实，城乡居民最低生活保障标准提高15%，城乡居民基础养老金提高28.8%，城镇职工退休人员基本养老金提高5.4%。新建村级公共服务中心66个，建成技艺传承场馆6个，8个项目入选广西“非遗”名单，成功举办或承办省级以上重大体育赛事19项。顺利完成创建广西食品安全示范城市考评验收。“平安梧州”建设深入推进，成功侦破一批涉黑涉恶大案要案，扫黑除恶专项斗争成效指标得分排名全区第二名，群众安全感由上年全区第九名提升到第六名。

（八）抓效能，强肌体。坚持依法全面履行政府职能，“法治梧州”建设深入推进，“七五”普法工作取得新成效。加强政府立法工作，出台《梧州市餐厨垃圾管理办法》等政府规章，推动出台《梧州市停车场建设和管理条例》等地方性法规。自觉接受人大监督和政协民主监督，人大代表建议和政协委员提案办结率达到100%。全面推进政务公开。加强行政复议和行政应诉工作。推动公共资源集中交易，全年各类公共资源交易节约资金2.26亿元。认真落实党风廉政建设主体责任，政府系统党风廉政建设和反腐败工作取得新成效。大力倡导“实干、干实、快干、干好”工作作风，政府工作效能得到提升。

一年来，国防动员、双拥共建、优抚安置、人民防空工作深入开展，妇女儿童、老年人和残疾人权益得到有效保障，编制、民族宗教、外事侨务、港澳台、审计、统计、机关事务、住房公积金、物价等工作取得新成绩，工会、共青团、科协、社会科学、慈善、档案、地方志等工作迈出新步伐，中直、区直驻梧各单位为全市经济社会发展做出了积极贡献。

过去一年，在极其困难的情况下，我们取得的成绩来之不易。这些成绩是自治区党委、政府和市委正确领导的结果，是市人大和市政协有效监督、大力支持的结果，是全市干部群众团结拼搏、克难攻坚的结果。在此，我代表市人民政府，向人大代表、政协委员，向各民主党派、工商联、无党派人士、人民团体和社会各界人士，向离退休老领导、老同志，向驻梧部队、武警官兵、公安干警，向所有关心支持梧州发展的朋友们，表示衷心的感谢和崇高的敬意！

过去一年，尽管我们付出了艰辛努力，但规模以上工业增加值出现了负增长，导致个别经济指标没有实现预期。出现这种状况，一方面是我们主动适应新时代高质量发展要求，加大转方式、调结构的力度，淘汰了一批落后产能；另一方面，多年来我市工业发展积累了一些结构性矛盾，工业经济进入了调整期。这是我市经济发展过程中阶段性、特殊性和暂时性的现象，只要我们坚定信心、保持定力、积极应对，全市经济社会发展长期向好的基本面是不会改变的。

我们清醒地认识到，当前我市还有不少问题和困难。主要是：发展新动能成长缓慢，产业结构不优，地方财力薄弱，县域经济不强，城乡发展不平衡，民生欠账较多，干部作风建设还需进一步加强，等等。对此，我们一定高度重视，认真加以解决。

二、2019年主要任务和重点工作

今年是中华人民共和国成立70周年，是全面建成小康社会关键之年，做好全年工作意义重大，影响深远。

今年政府工作的总体要求是：以习近平新时代中国特色社会主义思想为指导，按照建设壮美广西、共圆复兴梦想的总目标总要求，坚持新发展理念，坚持稳中求进工作总基调，坚持推动高质量发展，以供给侧结构性改革为主线，大力弘扬“海纳百川、力争上游”新时代梧州精神，毫不动摇实施“东融”战略，统筹抓好稳增长、促改革、调结构、惠民生、防风险、保稳定各项工作，持续打好三大攻坚战，保持经济持续健康发展和社会大局稳定，为全面建成小康社会收官打下决定性基础，以优异成绩庆祝中华人民共和国成立70周年。

今年经济社会发展的主要预期目标是：地区生产总值增长7%左右，财政收入增长5%，规模以上工业增加值增长5.7%，固定资产投资增长10%，社会消费品零售总额增长10%，外贸进出口总额增长3%，节能减排降碳控制在自治区下达目标内，居民人均可支配收入实际增长7%左右，城镇登记失业率控制在4.5%以内，居民消费价格涨幅3%左右，农村贫困人口减少5.5万。

实现上述总体目标，必须做好打硬仗的充分准备，我们将重点做好以下工作：

（一）聚焦产业提质，着力巩固稳中向好态势。以工业发展为主攻方向，精准施策，全力以赴稳住和提升经济基本面。

大力推动工业高质量发展。围绕强龙头、补链条、聚集群的要求，加快传统产业“二次创业”，推动战略性新兴产业倍增发展和轻工业振兴发展，加快谋划和形成200亿元级、300亿元级、500亿元级的现代工业体系梯度发展格局。以补链条、强配套为重点，大力推动生物医药、再生资源、再生不锈钢、林产林化、陶瓷、钛白、石材等产业向精深加工方向和产业链高端延伸。以扩能改造为重点，全力整合提升日用化工、五金水暖、纺织服装等轻工产业，再创梧州轻工业发展新优势。以培育产业集群为重点，推进机械制造、电子信息、生物制药、食品加工等产业优化升级。继续安排1亿元工业高质量发展专项资金，重点支持产业技改项目，支持停产、半停产企业并购重组，鼓励企业扩大产能，扶持企业家成长。实施大企业成长工程，重点支持中恒、神冠、五一、金升、永达等龙头企业发展壮大，加快培育一批产业领军企业。

大力推动产业园区提档升级。完善园区管理体制，探索“大园带小园”“园区+基地”等多元开发运营模式，加大园区基础设施投入，加快建设一批标准厂房。加快推动梧州高新区升格为国家级高新区、循环经济产业园区创建国家再制造产业示范基地、临港经济区创建自治区级开发区，加快再生不锈钢制品产业园生态化改造，加快岭南食品工业小镇（产业园）、生物医药产业园、轻工产业园、钛白产业园、生态铝产业园、六堡茶产业园、岑溪市稀土新材料科技产业园等特色园区建设。支持各县（市、区）结合各自资源条件，建设工业集中区、特色工业小镇，加快县域经济发展壮大。突出园区招商主体，大招商、招大商，实现产业招商大突破，以大项目支撑园区大发展，加快打造产值超1000亿元产业园区。

大力推动服务业提速发展。加快商贸物流园区、临港经济区、粤桂合作特别试验区广西现代服务业集聚区、科技服务业聚集区建设，大力发展港口物流等生产性服务业。实施电子商务“十百千万”工程，加快电子商务在线交易类、营销服务类、物流服务类等电子商务平台发展。加快大数据清洗加工基地建设，加快发展信息技术服务业。加快梧州恒大养生谷等项目建设，支持蒙山县创建国家级全域旅游示范区，实施骑楼城保护提升工程，打造岭南特色美食街，改造提升一批景区景点，全力打造岭南文化休闲旅游名城。大力发展养老、家政、教育培训等生活性服务业。改造提升批发零售业，推动农贸市场、商业综合体、特色街升级改造。抓好重点商品促销活动，提高六堡茶、龟苓膏等本地特色产品竞争力和市场占有率。抓住西江机场通航新机遇，筹划更多的全国性行业论坛、重大节会等在梧州举办，促进会展经济、商务经济加快发展。

更加精准高效服务企业。巩固“三去一降一补”成果，抓好降成本“41条”“28条”等措施落实。推动国家减税降费政策落地。扩大电力市场交易范围。进一步清理规范涉企行政事业性收费和经营服务性收费，下大气力帮助企业减负减荷、轻装上阵。完善市领导联系服务企业制度，持续推进企业上市（挂牌）“三大工程”，组织好政银企对接等活动，加大“小升规”政策支持，主动为企业排忧解难，推动一批企业复产、达产、稳产，确保新增规上工业企业40家以上。

大力扶持发展民营经济。实施强优民营企业培育计划，大力营造公平竞争环境，全面兑现政策承诺，集中梳理和解决历史遗留问题，切实维护民营企业合法权益。拓宽民营企业投资领域，鼓励民间资本参与国有企业混合所有制改造，鼓励民营企业参与PPP项目建设和运营。培育壮大民营企业家队伍，大力激发和弘扬企业家精神。

（二）聚焦“东融”发展，着力提升开放合作水平。毫不动摇实施“东融”战略，努力打造广西“东融”的枢纽门户城市。

加快粤桂合作特别试验区建设。巩固国家工业用地市场化配置改革试点成果，推动国家增量配电业务改革试点取得实质性成效，推动粤桂合作特别试验区列入国家级开发区名录。加快推进标准厂房、电网路网等基础设施建设。推动中锂日产20万支锂离子电池生产等项目开工，推进雅士东盟智能家居产业基地等项目建设，实现国光梧州产业基地、中盟中小企业园等项目竣工投产，力争新增规上工业企业10家，工业总产值、固定资产投资、招商引资到位资金分别增长12%、20%和15%。

实施产业大招商三年行动计划。坚持“一把手”招商，推进招商引资体制机制创新，打造招商引资项目代办服务、政企沟通、投诉举报三大平台，强化项目落地考核，力争招商引资到位资金增长8%以上。加大粤港澳大湾区重点城市商协会招商、产业链招商、驻点招商的力度，力争在重点产业领域招商实现新突破。积极开拓长三角、京津冀等招商“新战场”，全面对接生物医药、信息技术等高端产业外溢。加快循环经济园区、不锈钢制品园区、藤县高品质钢铁基地建设，积极承接京津冀钢铁产业转移。

大力推进多层次多区域合作。主动参与珠西经济带、南广高铁经济带、粤桂黔高铁经济带区域合作发展。健全西江经济带城市共同体及市长联席会议制度，推进与广州、佛山、肇庆、云浮、茂名等城市的务实合作。积极拓展与西江沿线港口城市合作，探索构建区域联动的内河港口运输体系。深化与港澳台的经贸文化交流合作。办好第四届广西（梧州）粤剧节、“岭南风情文化旅游梧茂交流年”、广西岭南风情文化旅游周、梧州宝石节、桂台茶产业发展论坛等活动。

大力促进外向型经济回暖。深入实施新一轮加工贸易倍增计划，加快粤桂合作特别试验区CEPA先行先试示范基地建设，推进西江集团梧州公司等外贸综合服务平台建设。支持宝石城、毅德城市场采购贸易试点，大力发展跨境电子商务等外贸新业态。全面落实外商投资企业商务备案与工商登记“一口办理”政策，深化口岸“单一窗口”建设，完成梧州港赤水作业区封关运行，巩固梧州至香港外贸航线，积极落实口岸提效降费各项政策措施。

（三）聚焦投资增效，着力增强发展支撑能力。发挥投资对稳增长基础性作用，力争年内完成固定资产投资560亿元。

抓好交通基础设施项目建设。加快推进柳州经梧州至广州铁路、洛湛铁路永州至玉林段电气化改造工程前期工作，积极争取南宁经玉林至深圳高速铁路规划建设经过岑溪。推进贺州至巴马、荔浦至玉林高速公路梧州段加快建设，争取梧州南绕城线（题甫至六坊段）公路等开工建设，加快推进梧信高速公路建设，建成G207东绕城过境公路、G355梨埠至马江二级公路。持续推进“四好农村路”建设，推动村级路网升级，力争苍梧县、蒙山县、万秀区、长洲区创建成为自治区级“四好农村路”示范县。加快西江黄金水道建设，力争建成大利口码头3—4号泊位，加快推进贵港至梧州3000吨级航道工程。积极培育梧州西江机场新航线，加快建设梧州通用航空基地。

抓好城镇基础设施项目建设。按照整体规划、分步推进的原则，科学推动城镇基础设施建设和城市新区发展。实施城市基础设施整体提升工程，打通一批“断头路”，修复一批破损路，畅通市区道路“微循环”。实施城市美化亮化工程，改造一批公园绿地、建设一批城市绿道。加快推进工厂一路、富民片区、河东片区等棚户区改造。加快苍海新区产城融合发展，加快环城水系、皇冲顶森林公园等项目建设。全面提升红岭新区综合承载力。加快推进城区防洪排涝工程和地下管网整治工程建设。加快苍梧新县城建设，推进岑溪新型城镇化试点县建设，推动藤县县城与梧州市中心城区同城化发展。抓好苍梧六堡特色小镇和蒙山丝艺小镇建设，推动百镇建设示范工程。

抓好农村基础设施项目建设。大力实施农村基础设施、公共服务能力“双提升”行动。加快实施自然村屯硬化路工程，完成农村道路“窄改宽”目标。全力做好“第一公里·路桥”惠民工程收尾工作。实施农村饮水安全巩固提升工程，推进一批防洪工程、农田水利、中小河流治理项目建设。实施新一轮农村电网升级改造，推进“县县通天然气”和天然气进乡入村。加快推动光缆、光纤网络向行政村户纵深延伸。健全县乡村三级医疗卫生机构分工协作机制和公共文化体育设施体系。

抓好项目建设推进机制创新。强化项目前期工作，争取将更多的项目纳入国家基础设施补短板、乡村振兴等相关规划。积极谋划全区产业项目观摩会及重大项目集中开竣工等活动。创新项目招投标、财评、征地拆迁等工作模式，大力破解项目建设瓶颈问题。努力扩大民间投资，在审批许可、经营运行等方面降低准入条件，大力创新投融资模式，为民营企业创造公平竞争环境和充足市场空间，鼓励民间资本扩大投资领域。

（四）聚焦改革创新，着力汇聚发展新动能。坚持改革创新引领，统筹推进、重点突破，破解发展难题。

全力优化营商环境。开展优化营商环境重点问题集中整改行动，实施营商环境评价指标提升方案，建立营商环境状况季度红黑榜预警制度。全力推动“354560”改革，推进项目审批制度改革，推广投资项目承诺制。加快建设公共大数据平台，破解信息孤岛，全面深化“最多跑一次”改革。探索受理与办理、办理与监督分离，深入推行“双随机一公开”，做到审批更简、监督更好、服务更优。加快跨部门、跨层级“一件事情”梳理归集，拓展“一事联办”范围，力争在商事登记、“证照分离”、不动产登记等领域改革取得突破，力争实现1个工作日完成企业开办。

大力推动重点领域改革。统筹抓好政府机构改革，优化机构设置与职能配置。加快推进“多规合一”改革，推动发改、国土、环保、城建、交通等规划有机整合。改进耕地占补平衡管理，健全耕地保护补偿机制。进一步深化国资国企改革。深化财政改革，实施全面预算绩效管理。积极推进城市管理执法、环卫、绿化等管理体制机制改革创新，提升城市精细化管理水平。深化农村领域改革，重点推进农村集体产权制度、农村土地“三权分置”改革。统筹抓好统计、安全生产、医药卫生、供销社、社保费和非税收入征管等改革。

强化科技创新引领。实施科技创新支撑产业高质量发展行动，力争新组建自治区级工程技术研究中心2个、科技企业孵化器1个，全市高新技术企业保有量达到75家，新增科技成果转化25项以上。实施“名校、名院、名所、名人”培育引进工程，大力引进、培育一批科技领军人物、青年科技人才、创新创业团队和紧缺适用高层次人才。推进大数据产业（梧州）基地、政务数据灾备中心等建设，打造数字经济新引擎。

（五）聚焦乡村振兴，着力开创“三农”新格局。坚持农业农村优先发展，加快实施乡村振兴战略。

加快发展现代特色农业。实施现代特色农业高质量发展三年提升行动，推动富硒稻米、六堡茶、果蔬、食用菌、优质畜禽、蜂蜜、桑蚕等特色农产品品牌创建、申报“三品一标”，加快推进“蜂情小镇”“蜜意小村”建设。深入推动现代特色农业示范区增点扩面提质升级，创建自治区级核心示范区3个、县乡村三级示范点352个，力争创建1个以上自治区五星级示范区。大力培育农业企业、农民合作社和家庭农场等新型农业经营主体。实施化肥农药使用量零增长行动。推广“微生物+”生态养殖模式。继续开展土地整治和高标准农田建设，稳定粮食综合生产能力。加快农业机械化和农机装备升级。

加快发展农产品加工等多元农业产业。实施农产品加工提升行动，加强农产品冷链物流体系和网点建设，加快建设六堡茶加工园区、岭南食品工业小镇（产业园）等园区建设，提高主产区农产品就地加工率。大力扶持推广农业“互联网+”模式，支持供销、物流、金融等企业把服务网点延伸到乡村。大力发展休闲农业和乡村旅游，提质升级“梧州六堡茶”公共品牌及“茶船古道”文化品牌，创建一批农村融合发展示范县、打造一批田园综合体、茶旅综合体。

大力建设“美丽梧州·幸福乡村”。巩固清洁乡村、生态乡村、宜居乡村建设成果，实施乡村风貌提升三年行动，开展“厕所革命”、污水治理等六大行动，探索农村垃圾分类处理模式，集中打造一批自治区级村屯绿化美化景观提升示范村。推动乡村规划全覆盖，加大农村“空心房”、危旧房改造力度，盘活农村宅基地资源，规范农村建房，塑造农村建筑新风貌。

（六）聚焦绿色发展，着力提升生态文明水平。做好生态文明建设加减法，不断推动环境保护与经济社会发展融合共进。

坚决打好污染防治攻坚战。切实抓好中央环保督察“回头看”反馈问题整改落实。坚决打好“蓝天”保卫战，推进工业企业污染综合治理，杜绝“散乱污”项目建设；深入实施大气污染防治综合治理行动，狠抓市区烟花爆竹禁燃禁售和污染源头管控。坚决打好“碧水”保卫战，加强饮用水水源保护区环境综合整治，加快推进城市取水口上移工程、污水直排口治理工程和第三污水处理厂建设，完善城镇生活污水处理设施建设，实现工业集聚区污水集中处理设施全覆盖，全面完成县级饮用水水源地问题整治。坚决打好“净土”保卫战，加强农业面源污染防治、涉重污染排查整治和废弃矿山生态修复，严厉打击跨区域非法转移倾倒固废危废行为。全面完成第二次全国污染源普查和第三次国土调查任务。

大力发展绿色经济。加快新型生态产业发展，推动再生不锈钢、林产林化等传统资源型产业改造提升，推进资源型园区开展清洁能源替代，确保主要工艺指标和生产设备效能达到国家标准。大力发展绿色、有机农产品，发展生态综合种养和林下经济。推广绿色建筑，倡导绿色出行。开展“美丽梧州”国土绿化三年行动，加快推进苍海国家湿地公园、梧州广信森林公园等生态公园建设，推进西江沿岸生态农业带建设，筑牢西江绿色屏障。

健全环境保护制度体系。强化土地用途管制和生态红线管理，全面开展“大棚房”清理整治，突出绿色发展绩效评价考核。探索建立以排污许可证为核心的环境监管新模式，建立环境保护“红黄牌”管理制度，完善企业环保失信联合惩戒机制。推进环保机构监测监察执法垂直管理制度改革，加大环境违法案件处置力度，严厉打击环境违法行为。

（七）聚焦民生福祉，着力增强群众获得感。以脱贫攻坚和补齐民生短板为重点，加大民生投入，不断增进民生福祉。

打好脱贫攻坚战。紧扣“两不愁三保障”，聚焦深度贫困地区和特殊贫困群体，注重脱贫质量，深入推进“打赢脱贫攻坚战三年行动”。进一步压实脱贫攻坚责任，抓好就业扶贫、综合保障性扶贫等“五大专项行动”，打好产业扶贫、易地扶贫搬迁等“五场硬仗”，确保实现5.5万人脱贫、70个贫困村出列、藤县脱贫摘帽。开展对已脱贫摘帽县“回头看”，切实抓好扶贫专项巡视发现问题整改。

加强就业和社会保障。狠抓高校毕业生、农民工、就业困难人员等重点群体就业创业，力争城镇新增就业2.5万人，农村劳动力转移就业4.5万人次。建立健全解决拖欠农民工工资问题的长效机制。持续夯实基础养老工作，全面实施全民参保计划，确保各项社保待遇按时足额发放。加强城乡社会救助体系建设。加强优抚安置工作，维护军人军属合法权益，促进军民融合发展，全力创建全国“双拥模范城”。

深入实施教育提升三年行动计划。优化教育资源布局，新建、改扩建公办幼儿园10所，改造农村义务教育学校88所，确保各县（市、区）全部通过国家义务教育均衡发展评估。实施高中阶段教育攻坚计划，推行新高考改革，提高高中教育质量。大力支持和规范民办教育，推行职业教育集团化办学，促进校企合作办学和中高职教育有机衔接。加快梧州医学高等专科学校建设，大力提升梧州职业学院办学水平，全力支持梧州学院加快建设特色鲜明的应用型高水平大学。实施教师队伍提质增量工程，落实中小学校人才激励办法和乡村教师支持计划。

提升卫生健康事业发展水平。加快工人医院、妇幼保健院等改扩建项目和市120急救指挥中心建设。推进医联体和县域医共体建设，完善分级诊疗制度。深入推进基层医疗卫生机构能力建设行动计划，加快标准化村卫生室建设。大力发展康养产业，实施“智慧医疗”二期工程，深入开展“互联网+医疗健康”便民惠民活动。加强全科医生队伍建设，支持特色专科发展，推动中医药事业传承。加强重大疾病防控。优化计划生育管理服务。巩固广西食品安全示范城市创建成果。全力创建国家卫生城市。

大力发展文化体育事业。围绕庆祝中华人民共和国成立70周年开展系列文化体育活动。积极开展群众性精神文明创建活动，加快创建全国文明城市。加快梧州书城、县（市）图书馆、融媒体中心等项目建设。加强优秀文化传承和文化遗产保护。扎实推进农村文化惠民项目建设。深入实施体育惠民工程，加快推进新体校建设，广泛开展全民健身活动。举办梧州首届市民运动会，积极备战第十四届广西运动会，筹备广西第十届残疾人运动会。

加强和创新社会治理。打好防范化解重大风险攻坚战，坚决遏制政府隐性债务增量，妥善化解存量，加强地方金融监管和风险监测预警，坚决打击违法违规金融活动，确保不发生系统性区域性金融风险。加大城乡社区治理力度，完善村（居）民自治体系建设。加强行政复议、行政调解和人民调解工作。严格落实安全生产责任制，守住安全生产底线。持续推进“法治梧州”“平安梧州”建设，深入开展扫黑除恶专项斗争。做好第四次全国经济普查。做好民族宗教、外事侨务、保密、港澳台、统计、审计、人防、气象、地震、档案、地方志、老龄、机关事务、救灾救济和慈善等工作，支持工会、共青团、妇联、工商联、残联等群团组织发挥更大作用。

各位代表，保障和改善民生是我们发展的根本目的。今年我们将筹措资金50亿元以上，继续实施社保、健康、教育、水利、安居、农补、生态、扶贫、文化、科技等一批为民办实事项目，让人民群众获得感更加充实！

三、加强政府自身建设

实现宏伟蓝图，贵在务实重行。我们将牢记人民重托，敢担当、善作为，真抓实干、埋头苦干，全面提升施政能力和服务水平。

（一）履职尽责重法治。严格按照法定权限和程序用权履职，确保政府工作在法治轨道上运行。自觉接受人大监督和政协民主监督，提高办理人大代表建议和政协提案的落实率和满意率。加强在保障公民权利、优化营商环境、环境保护、城市管理等领域的政府立法，提高立法质量。健全重大行政决策合法性审查机制，坚持科学决策、民主决策、依法决策，主动接受社会监督，推进审计监督全覆盖，加大政务公开力度，主动回应社会关切，让权力始终在阳光下运行。

（二）开拓创新争上游。增强坐不住、等不起的紧迫感和危机感，在抓机遇、搭平台、补短板、推动梧州高质量发展上，勇挑重担、敢闯新路，让“向高的攀、跟强的比、与快的赛”成为政府工作的风向标。

（三）真抓实干敢担当。大力弘扬“实干、干实、快干、干好”的工作理念和作风，坚持解放思想、改革创新，尽责履职、躬身前行，在工业发展、项目建设、产业大招商、优化营商环境等问题上，敢于较真碰硬，大力破解“中梗阻”问题。说了的坚决做到、定了的坚决执行，真正把责任扛到肩上、工作落到实处，让担当作为成为政府工作的主旋律。

（四）心存敬畏守底线。坚定不移把党的政治建设摆在首位，深入推进政府系统党风廉政建设和反腐败工作，进一步转变作风，坚决纠正不作为、慢作为等现象，严惩懒政怠政、失职渎职行为，努力营造风清气正的政治生态。

各位代表！新时代开启新征程，新时代呼唤新作为。让我们更加紧密团结在以习近平同志为核心的党中央周围，在自治区党委、政府和市委的坚强领导下，认真践行“担当为要、实干为本、发展为重、奋斗为荣”为政理念，大力弘扬“海纳百川、力争上游”新时代梧州精神，坚定信心，砥砺前进，为建设壮美广西、共圆复兴梦想贡献梧州力量，以优异成绩庆祝中华人民共和国成立70周年！
